# Hybrid Nanoparticle-Assisted Chemo-Photothermal Therapy and Photoacoustic Imaging in a Three-Dimensional Breast Cancer Cell Model

**DOI:** 10.3390/ijms242417374

**Published:** 2023-12-12

**Authors:** Barbara Carrese, Chiara Cavallini, Paolo Armanetti, Brigida Silvestri, Gaetano Calì, Giuseppina Luciani, Gennaro Sanità, Luca Menichetti, Annalisa Lamberti

**Affiliations:** 1 Department of Molecular Medicine and Medical Biotechnology, University of Naples Federico II, 80131 Naples, Italy; 2 Institute of Clinical Physiology, National Research Council, 56124 Pisa, Italy; 3 Department of Civil, Construction and Environmental Engineering, University of Naples Federico II, 80125 Naples, Italy; 4 Institute of Endocrinology and Molecular Oncology, National Research Council, 80131 Naples, Italy; 5 Department of Chemical, Materials and Production Engineering, University of Naples Federico II, 80125 Naples, Italy; 6 Institute of Applied Sciences and Intelligent Systems—Unit of Naples, National Research Council, 80131 Naples, Italy

**Keywords:** albumin-modified nanoparticles, melanin, doxorubicin, breast cancer spheroids, photoacoustic imaging, photothermal therapy

## Abstract

Bioinspired nanoparticles have recently been gaining attention as promising multifunctional nanoplatforms for therapeutic applications in cancer, including breast cancer. Here, the efficiency of the chemo-photothermal and photoacoustic properties of hybrid albumin-modified nanoparticles (HSA-NPs) loaded with doxorubicin was evaluated in a three-dimensional breast cancer cell model. The HSA-NPs showed a higher uptake and deeper penetration into breast cancer spheroids than healthy breast cell 3D cultures. Confocal microscopy revealed that, in tumour spheroids incubated with doxorubicin-loaded NPs for 16 h, doxorubicin was mainly localised in the cytoplasm, while a strong signal was detectable at the nuclear level after 24 h, suggesting a time-dependent uptake. To evaluate the cytotoxicity of doxorubicin-loaded NPs, tumour spheroids were treated for up to 96 h with increasing concentrations of NPs, showing marked toxicity only at the highest concentration of doxorubicin. When doxorubicin administration was combined with laser photothermal irradiation, enhanced cytotoxicity was observed at lower concentrations and incubation times. Finally, the photoacoustic properties of doxorubicin-loaded NPs were evaluated in tumour spheroids, showing a detectable signal increasing with NP concentration. Overall, our data show that the combined effect of chemo-photothermal therapy results in a shorter exposure time to doxorubicin and a lower drug dose. Furthermore, owing to the photoacoustic properties of the NPs, this nanoplatform may represent a good candidate for theranostic applications.

## 1. Introduction

Breast cancer (BC) is one of the most dangerous female tumours; in 2020, about 2.3 million women were diagnosed with breast cancer, and in the same year, 685,000 deaths were recorded [1]. This disease is treatable in a high percentage of patients (~70–80%) who receive an early diagnosis before metastasis occurrence [1,2,3], and, for this reason, a rapid diagnosis and an effective, precise therapy are needed [4]. Among the various types of breast cancer, triple-negative breast cancer accounts for about 10–15% of cases, and it is the one with the worst prognosis. This tumour, which does not express oestrogen and progesterone receptors or Human Epidermal Growth Factor Receptor 2 [5], is resistant to receptor-targeted therapies, and chemotherapy remains the only possible option [6].

The anticancer drug doxorubicin (DOX) is one of the most potent drugs used in the clinic as a chemotherapeutic agent to treat various cancers, including BC [7,8]. However, the clinical application of DOX is limited by its harmful side effects, such as cardiotoxicity, neutropenia, neuropathy, anaemia, nephrotoxicity, and hepatotoxicity, due to its inability to discriminate cancer cells from normal cells [9]. Overcoming DOX side effects is the greatest challenge for its clinical application, and efforts to develop new drug delivery nanocarriers for drug delivery address this scenario.

A valuable option to counter the harmful effects of chemotherapeutics is to encapsulate them into selective carriers, such as nanoparticles. One of the most used nanoparticles for DOX-mediated cancer therapy is pegylated liposomal doxorubicin (PLD), approved for clinical usage in November 1994 [10]. Until today, many new types of nanoparticles have been developed to significantly improve the clinical use of DOX and reduce its side effects through the enhanced permeation and retention (EPR) effect and/or active targeting delivery [9,11]. Nanoparticles also allow for combining traditional therapeutic strategies with emerging therapeutic approaches, such as photothermal therapy (PTT) [12,13,14,15].

PTT uses photosensitisers (PSs), including plasmonic nanoparticles, to generate heat at the tumour site following the absorption of near-infrared (NIR) light [16]. The resulting local hyperthermia promotes cell death, sensitises tumour cells to additional treatments such as chemotherapy and radiotherapy [17], and enhances drug delivery [18]. Since NIR light is safe and adjustable to meet therapeutic needs [19], and nanoparticles can be designed to increase their tumour-targeting ability, nanoparticle-assisted PTT holds the potential to treat cancer precisely, non-invasively, and with few side effects [20]. Moreover, nanoparticles used as PSs can also act as contrast agents for photoacoustic imaging [16,17,18], a non-ionising, non-invasive, high-resolution technique which combines optical imaging and ultrasounds. Thus, suitable nanoparticles enable the combination of chemo-photothermal therapy and diagnostic properties, paving the way for theranostic applications [19,20,21].

In recent years, drug screenings and studies of tumour growth and proliferation, as well as property assessment of new drug/contrast agent (CAs) delivery systems, have been based on the use of three-dimensional (3D) culture methods, which could serve to bridge the gap between conventional 2D cell cultures and animal models. 3D cultures, such as spheroids, mimic the in vivo tumour microenvironment through the secretion of their own extracellular matrix (ECM) and the formation of cell–cell interactions [21,22], which are absent in two-dimensional monolayer cell culture systems, and thus represent an essential tool for optimising efficient intratumoral drugs/CAs delivery.

Recently, we developed hybrid eumelanin–silver NPs (MelaSil_Ag) with photoacoustic properties [23], functionalised with human serum albumin (HSA), targeting breast cancer cells via HSA–SPARC interaction [24], to design DOX carriers (MelaSil_Ag-HSA@DOX NPs) for BC treatment. MelaSil_Ag-HSA@DOX NPs demonstrated promise as a platform for an integrated photothermal (PT) chemotherapy approach in a 2D model [14,24]. Here, the photoacoustic and chemo-photothermal properties of these nanoparticles were evaluated in a 3D BC culture model to confirm the efficacy of this novel nanoplatform in a system that mimics the in vivo tumour microenvironment.

## 2. Results and Discussion

### 2.1. NPs Characterisation

The hydrodynamic diameter (DH) and surface ζ-potential were assessed through DLS analysis. The average DH of MelaSil_Ag-HSA@DOX (DOX@NPs) nanoparticles was 407 nm with a polydispersity index (PDI) of 0.45. This size is typically not significant for drug delivery as the EPR effect usually operates in the range of 100–400 nm [25]. Although the desired nanoparticle size ranges from 10 to 200 nm, on the base of in vivo studies, there seems not to be a most effective specific size. Size is relevant not only for the efficiency of cellular uptake, type of internalisation pathway, and intracellular localisation, but also for in vivo biodistribution. In this view, a compromise between nanoparticles that are not too small, because this would make their permanence in the tumour difficult, and not too large, which could delay their internalisation into the cells, seems ideal. It is reported that particles ranging between 50 and 400 nm can be retained in tumours, while those from 5 to 50 nm can escape from the tumour area [26]. Recently, some works on doxorubicin-loaded nanoparticles with a size over 300 nm have been reported for breast cancer therapy [27].

Furthermore, the nanoparticles exhibit a negative ζ-potential (−17 ± 2.16 mV) due to the presence of negatively charged HSA on the NPs’ surface [28]. The size and ζ-potential of MelaSil_Ag (bare-NPs) and MelaSil_Ag-HSA (HSA-NPs) were also determined (Table 1).

**Figure 1:**
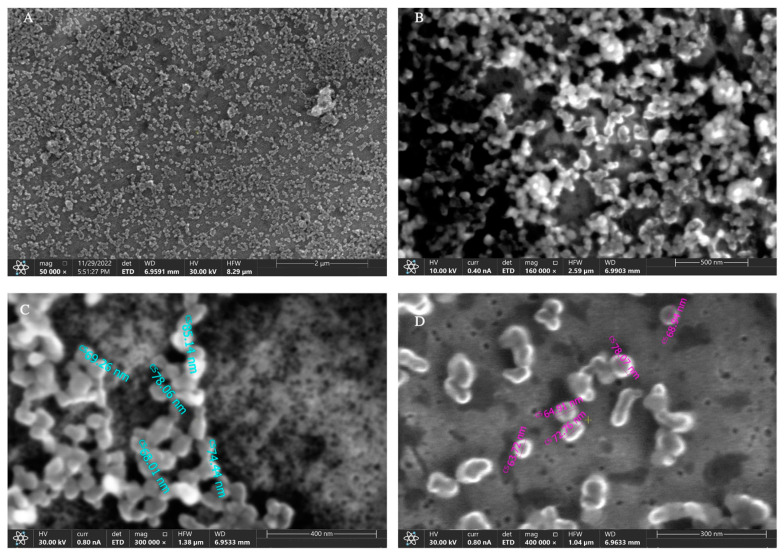
Representative images of DOX@NPs SEM analysis at different magnifications: (**A**) 50,000×; (**B**) 160,000×; (**C**) 300,000×; (**D**) 400,000×.

The SEM images (Figure 1) show a pseudo-spheric shape of the nanoparticles, with a size between 65 nm and 85 nm. The difference in size obtained by SEM analysis compared to DLS analysis could be related to the different conditions of the two techniques (dried and wet, respectively) and, therefore, to the dehydration of the sample for SEM acquisition, which can deconstruct the protein component represented by albumin conjugated to NPs surface.

### 2.2. Evaluation of Bare- and HSA-NP Uptake in Hs578T and MCF10a Spheroids

As experimental models, a triple-negative breast cancer cell line (Hs578T cells) and a mammary breast fibrocystic disease cell line (MCF10a) were used.

Hs578T cells were chosen because triple-negative breast cancers cannot be treated with endocrine therapy or therapies targeted to human epidermal growth factor receptor type 2; therefore, chemotherapy, limited by its harmful side effects, remains the only option. For this reason, preclinical research has made much effort to develop new drug delivery systems to overcome some limitations related to triple negative breast cancer therapy. MCF10a were chosen because they represent a normal counterpart of breast cancer cells, not showing any characteristics of invasiveness and not forming tumours when transplanted into immunodeficient mice. For this reason, their use in active targeting studies becomes mandatory.

**Figure 2:**
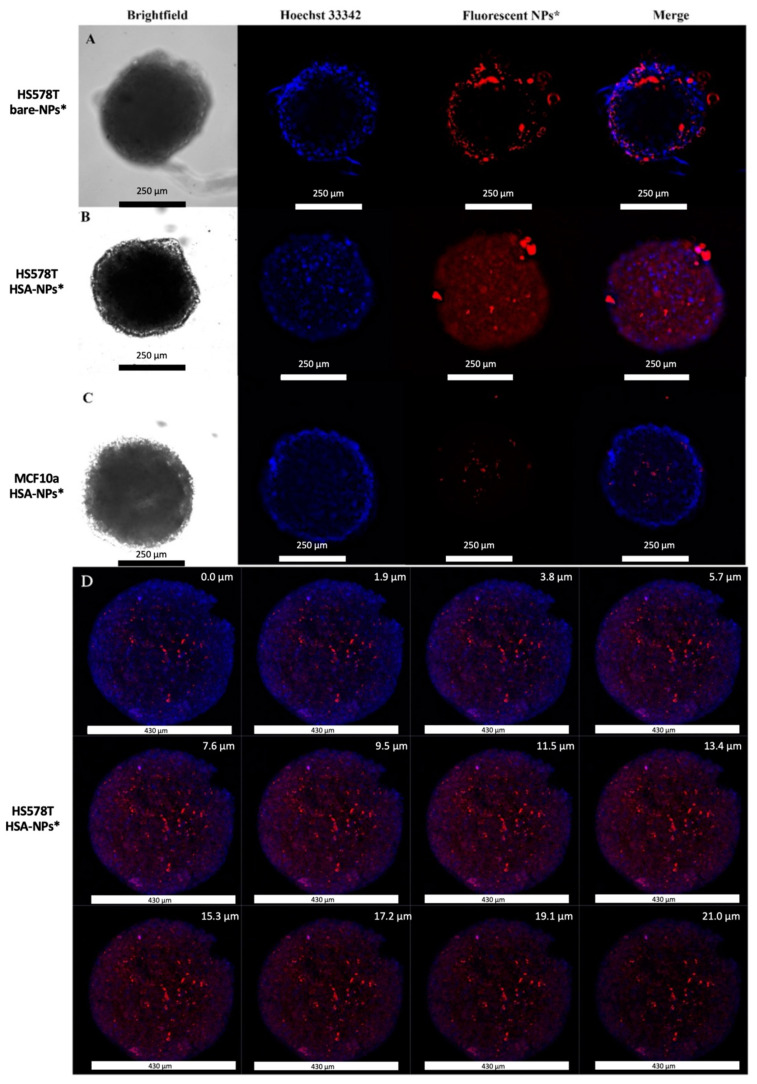
Representative images of (**A**) Thunder microscopy analysis of Hs578T spheroids treated with bare-NPs* for 18 h; scale bar: 250.0 µm; (**B**) Thunder microscopy analysis of Hs578T spheroids treated with HSA-NPs* for 18 h; scale bar: 250.0 µm; (**C**) Thunder microscopy analysis of MCF10a spheroids treated with HSA-NPs* for 18 h; scale bar: 250.0 µm; (**D**) confocal microscopy: gallery of merged images acquired along the z-axis of Hs578T spheroids treated with HSA-NPs* for 18 h; scale bar 430 µm. Cell nuclei were stained with Hoechst 33342 (blue color); bare- and HSA-NPs* are visible as red spots.

To evaluate HSA-NP uptake in Hs578T and MCF10a spheroids, Thunder light microscopy and confocal microscopy were performed. To this aim, Thunder microscopy was carried out by incubating Hs578T spheroids both with fluorescent rhodamine B bare-NPs* (Figure 2A) and HSA-NPs* (Figure 2B) at 200 μg/mL at 37 °C for 18 h. The tested concentration of nanoparticles is similar to that causing good tolerance when injected intravenously in nude mice [29]. Furthermore, MCF10a spheroids were also treated with HSA-NPs* (Figure 2C) in the same experimental conditions. The presented images, acquired by using the Thunder imaging system (z-stack), refer to the centre of each spheroid and clearly show that a greater amount of HSA-NPs was uptaken and entered deeper into the Hs578T spheroids compared to bare-NPs, whereas in MCF10a 3D culture, no internalisation was observed. Mean fluorescence intensity due to rhodamine B emission, calculated by using ImageJ, was 6.716 a.u. in Hs578T cells treated with bare-NPs*, 26.320 a.u. in Hs578T cells treated with HSA-NPs*, and 2.991 a.u. in MCF10a treated with HSA-NPs*. No aspecific fluorescence signal was detected in untreated control spheroids (Figure S1). This result may be attributed to secreted protein acidic and rich in cysteine (SPARC)-mediated internalisation in Hs578T cells (SPARC-positive) compared to MCF10a cells (SPARC-negative), as previously reported [24]. HSA-NP* internalisation in Hs578T spheroids was also evaluated by confocal microscopy (Figure 2D).

### 2.3. DOX Delivery in Hs578T Spheroids

**Figure 3:**
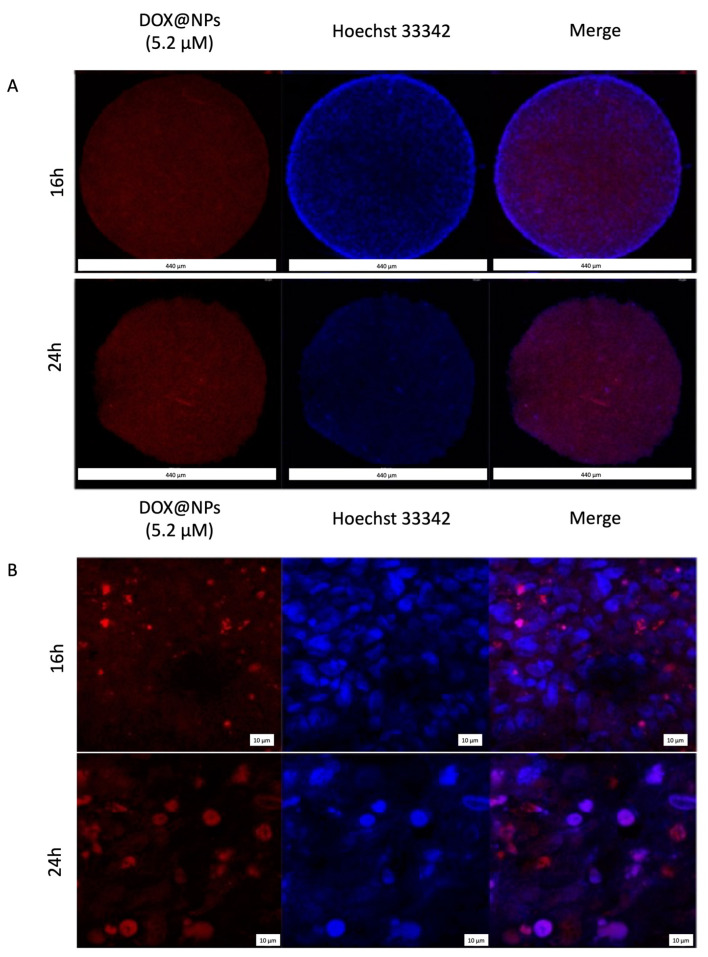
Representative image of z-stack confocal microscopy analysis of Hs578T spheroids treated with DOX@NPs at 200 µg/mL (DOX 5.2 µM) for 16 and 24 h. Panel (**A**): whole spheroid z-stack (scale bar 440 μm); panel (**B**): high magnification detail (scale bar 10 μm). Cell nuclei were stained with Hoechst 33342 (blue color); DOX is visible as red colour.

To evaluate the nuclear localisation of the drug delivered by DOX@NPs, confocal microscopy was performed. To this aim, Hs578T spheroids were treated with DOX@NPs at 200 μg/mL (with a corresponding amount of DOX of about 5.2 μM) at 37 °C for 3 h, 6 h, 16 h, and 24 h. Images (Figure 3) show that, after 16 h, DOX delivered by NPs was present in the cytoplasm while, after 24 h, an intense red signal at the nuclear level was visible, confirming that the internalisation of the drug carried by the NPs was time-dependent. Contrary to what was observed in 2D culture [14], no presence of DOX was observed after 3 and 6 h of incubation. This late kinetics was probably caused by the reduced access of nanoparticles into the spheroids due to the presence of the extracellular matrix (ECM) [30].

### 2.4. Light Microscopy Analysis of Hs578T Spheroids after DOX@NPs Treatment

**Figure 4:**
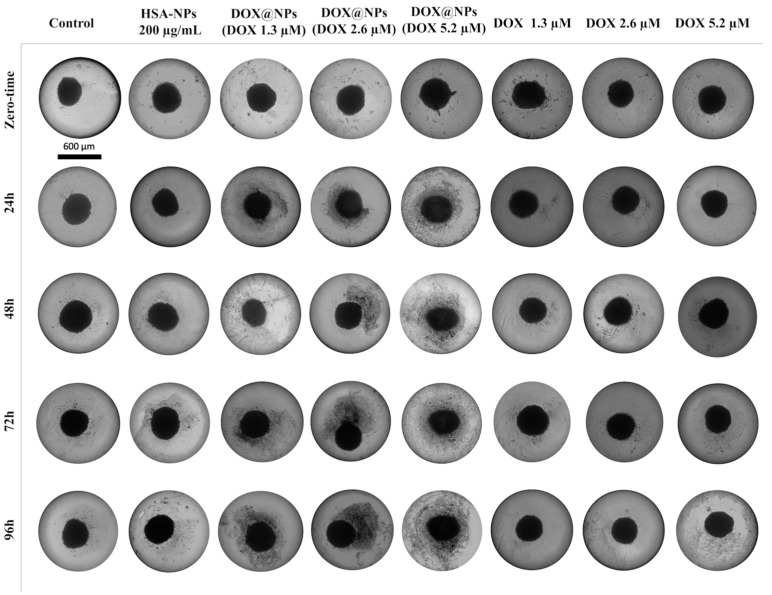
Light microscopy analysis of Hs578T 3D cell model treated with DOX@NPs and free DOX, scale bar: 600 µm.

To evaluate the effect of DOX delivered by DOX@NPs compared to free DOX, light microscopy analysis was carried out. Hs578T spheroids were treated both with HSA-NPs at 200 µg/mL as a negative control, and with DOX@NPs at increasing concentrations (50 µg/mL, 100 µg/mL, and 200 µg/mL corresponding to DOX amounts of about 1.3 µM, 2.6 µM, and 5.2 µM) and with the free drug in the same amount up to 96 h (Figure 4). Results show the presence of live cells around the spheroid, owing to their translucency, in the control group. In contrast, treatment with DOX@NPs induces the appearance of more cellular debrides around the spheroids in a time- and dose-dependent fashion compared to the free drug. This result is probably due to a SPARC-mediated internalisation of DOX@NPs that allows for the overcoming of multi-drug resistance (MDR) and for drug release into the spheroid’s acidic microenvironment [31,32,33].

### 2.5. Cytotoxicity of DOX@NPs

**Figure 5:**
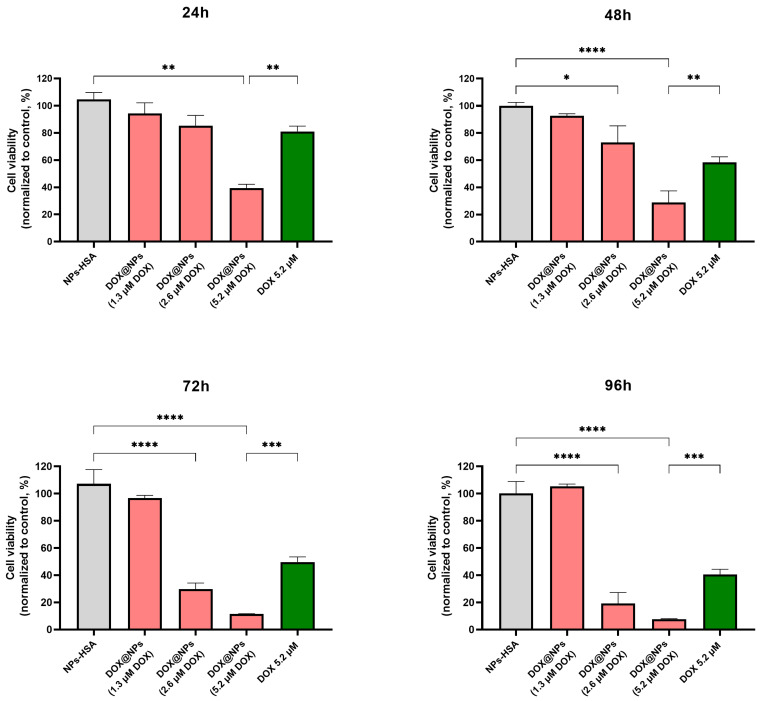
Cell viability assay of Hs578T spheroids treated for 24 h, 48 h, 72 h, and 96 h with NPs-HSA (200 μg/mL), DOX@NPs corresponding to amounts of DOX of about 1.3 μM, 2.6 μM, and 5.2 μM (DOX@NPs) at 50 μg/mL, 100 μg/mL, and 200 μg/mL, respectively) and with free DOX at 5.2 µM. * *p* ≤ 0.05; ** *p* ≤ 0.01; *** *p* ≤ 0.001; **** *p* ≤ 0.0001.

To evaluate the cytotoxicity of DOX@NPs in the Hs578T 3D model, a viability assay based on the quantitation of ATP, a marker for the presence of metabolically active cells (CellTiter-GLO 3D assay), was performed. Hs578T breast cancer spheroids were treated for up to 96 h with increasing concentrations of DOX@NPs (50 µg/mL, 100 µg/mL, and 200 µg/mL corresponding to DOX amounts of about 1.3 µM, 2.6 µM, and 5.2 µM), and with HSA-NPs at the highest concentration as control. Results (Figure 5) showed negligible toxicity when spheroids were treated with HSA-NPs and DOX@NPs at the lowest concentration (1.3 µM). At a DOX concentration of 2.6 µM, a viability decrease of about 15%, 27%, 70%, and 81% after 24 h, 48 h, 72 h, and 96 h, respectively, was detected. In comparison, at the maximum DOX concentration (5.2 µM), an appreciable viability decrease was already evident after 24 h of incubation (about 61%, 71%, 89%, and 92% after 24 h, 48 h, 72 h, and 96 h, respectively). Furthermore, the data were compared with results obtained in the 2D cell culture (Figure S2), showing that, up to 72 h, DOX@NPs, used at the same concentrations (DOX at 1.3 µM and 2.6 µM), caused very low toxicity in 3D cultures when compared to 2D cells. Particularly, to have comparable toxicity between the monolayer and the 3D model, it was necessary to use at least double the highest concentration or prolong the incubation time used for the 2D model. This result is unsurprising since cancer cells are less sensitive to drugs in 3D cultures than in 2D cultures. This effect could probably be due to reduced access to compounds in the medium or pathophysiological differences owing to hypoxia or changes in the cell cycle [34]. Furthermore, spheroids can simulate the drug resistance characteristic of solid tumours and thus require higher doses of drugs and longer incubation times than monolayer cell cultures [35,36]. Moreover, free DOX used at the highest concentration shows a lower toxicity when compared to that obtained by using DOX@NPs at the same DOX concentration. This effect was already observed in a 2D model and could probably be due to the overcoming of multidrug resistance when the drug is delivered by nanoparticles. As expected, no toxicity of DOX@NPs in MCF10a spheroids was observed (Figure S3).

### 2.6. Cytotoxicity of DOX@NPs Combined with Laser Irradiation

**Figure 6:**
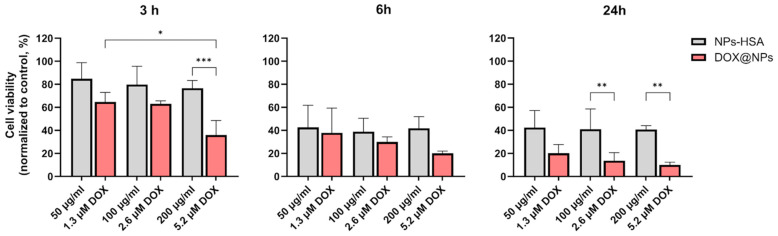
Cell viability assays of Hs578T 3D model following CW laser irradiation. CellTiter GLO assay of Hs578T spheroids treated for 18 h with DOX@NPs (red) or HSA-NPs (grey) at concentrations of 50 µg/mL, 100 µg/mL, and 200 µg/mL (corresponding to 1.3 µM, 2.6 µM, and 5.2 µM DOX for DOX@NPs) and irradiated for 5′ at 808 nm (3 W/cm^2^). * *p* < 0.05, ** *p* < 0.01, and *** *p* < 0.001. CellTiter-GLO assay was performed 3 h, 6 h, and 24 h after irradiation.

To explore the effect of NP administration in combination with laser photothermal irradiation, cell viability was evaluated at 3 h, 6 h, and 24 h after laser irradiation by quantifying the presence of ATP. To this aim, Hs578T spheroids were first incubated for 18 h with increasing concentrations of DOX@NPs or HSA-NPs and then irradiated with a continuous wavelength (CW) laser at 808 nm at 3 W/cm^2^ for 5 min. As reported previously [14], the plasmonic properties of MelaSil_Ag NPs provide a temperature rise of about 10 °C under CW laser illumination, which makes them suitable for photothermal treatment. As reported in Figure 6, the results show that combined chemo-photothermal treatment induces good cytotoxicity at lower DOX concentrations and incubation times compared to treatment without laser irradiation. In detail, 3 h post irradiation, spheroids treated with DOX@NPs showed a higher reduction in cell viability (about 32.00%, 37.00%, and 64.00% at 1.3 μM, 2.6 μM, and 5.2 μM of DOX, respectively) than that obtained without laser irradiation at the lowest concentration after 24 h of incubation (24 h, Figure 5). The contribution of the photothermal effect to cytotoxicity became even more evident 6 h and 24 h post irradiation, confirming the good photothermal properties of HSA-NPs in terms of heat delivery also on 3D models. In particular, 24 h post irradiation, cell viability was significantly reduced for DOX@NP-treated spheroids (80.00%, 86.00%, and 90.00% of cell viability reduction at 1.3 μM, 2.6 μM, and 5.2 μM of DOX, respectively) vs. about 10.00%, 27.00%, and 71.00% at the same DOX concentrations without laser irradiation after a comparable incubation time (48 h, Figure 5). These results highlight the excellent photothermal properties of MelaSil_Ag-HSA NPs that, when used without DOX, already after 6 h post irradiation cause a reduction in cell viability of about 57.94%, 58.00%, and 59.71% at 50 mg/mL, 100 mg/mL, and 200 mg/mL, respectively.

### 2.7. Photoacoustic Imaging of Hs578T Spheroids

**Figure 7:**
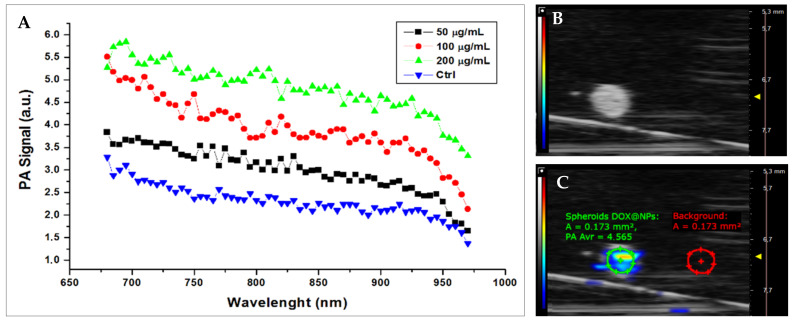
Photoacoustic multispectral response of Hs578T spheroids in optical near-infrared windows between 680 and 970 nm. (**A**) PA signal intensity (a.u.) at different laser wavelength stimulation. The graphic shows PA intensities at different wavelengths in the 680–970 nm range (step size 5 nm) of spheroids treated with DOX@NPs at increasing concentrations, from 50 μg/mL to 200 μg/mL, and the untreated control. (**B**) Representative B-Mode echographic image. (**C**) PA image of an Hs578T spheroid.

To verify the exploitability of DOX@NPs as photoacoustic probes in our 3D model, Hs578T spheroids were incubated with increasing concentrations of DOX@NPs and then analysed using high-frequency ultrasound/photoacoustic imaging. We were able to co-register the spectral features of DOX@NPs with corresponding imaging of high-frequency ultrasound, with a good detectability in terms of contrast-to-noise ratio (CNR) and signal-to-noise ratio (SNR), which were in the typical range (CNR: 12–35, SNR: 13–36, respectively). The photoacoustic signal intensity of Hs578T spheroids was compliant with the melanin absorption pattern, as previously studied [24], and was consistent with NP concentration, as shown in Figure 7A.

## 3. Materials and Methods

### 3.1. Materials

Doxorubicin HCl and recombinant human serum albumin were purchased from Sigma-Aldrich (St. Louis, MO, USA). Phosphate-buffered saline (PBS) was purchased from Gibco (Grand Island, NY, USA). BIOFLOATTM 96-well plate was obtained by faCellitate (Mannheim, Germany). Agar Bacteriology grade was purchased by Applichem (Darmstadt, Germany). Hoechst 33342 was purchased from Thermo Scientific™ (Waltham, MA, USA). CellTiter-GLO^®^ 3D Cell Viability Assay was purchased from Promega (Madison, WI, USA).

### 3.2. Nanoparticles Preparation and Characterisation

MelaSil_Ag (bare-NPs), MelaSil_Ag-HSA (HSA-NPs), and MelaSil_Ag-HSA@DOX (DOX@NPs) nanoparticles were synthesised and modified as already widely described in previous works [14,24,28]. The amount of DOX loaded to MelaSil_Ag-HSA NPs was calculated by taking advantage of the intrinsic fluorescence of DOX, as described in [14]. DOX bond efficiency and capacity were calculated for each preparation of MelaSil_Ag-HSA@DOX, and, on average, DOX concentration corresponding to 1 μg/mL of NPs was about 0.02 μM.

Size distribution and ζ-potential of nanoparticles were measured in PBS by a Zetasizer (Nanoseries, Malvern) using the laser dynamic scattering (λ = 632.8 nm) and particle electrophoresis techniques, respectively. For scanning electron microscopy (SEM), DOX@NPs samples were resuspended in bidistilled water, spotted on an aluminium sample holder, and dried at room temperature. SEM images were acquired using an ETD detector with different spot sizes and high voltage values.

### 3.3. Cell Culture

Triple-negative breast tumour cell line (Hs578T) and mammary breast fibrocystic disease cell line (MCF10a) were obtained from the American Type Tissue Collection (Rockville, MD, USA). Hs578T cells were grown in DMEM supplemented with 10% heat-inactivated foetal bovine serum (FBS) (GIBCO), 100 U/mL penicillin, 100 mg/mL streptomycin, 1% L-glutamine, and 0.01 mg/mL human insulin. MCF10a cells were grown in MEGM (Lonza, Basel, Switzerland) supplemented with a Mammary Epithelial Cell Growth Medium Bullet Kit (Lonza), 100 nM cholera toxin (Sigma-Aldrich), and 5% heat-inactivated foetal horse serum (FHS) (Lonza). All cell lines were grown at 37 °C in a 5% CO_2_ atmosphere.

### 3.4. Spheroid Formation

Hs578T spheroids were obtained by seeding cells at a density of 7500 cells/50 µL in ultralow attachment 96-well round-bottom plates. Every day, 20 µL of cell medium was replaced with fresh medium. After three days, 100 µL of fresh medium was added. MCF10a spheroids were obtained through the hanging drops method by seeding 7500 MCF10a cells in the form of 20 µL drops on the inverted lid of the 96-well plate. Every day, 5 µL of cell medium was replaced with fresh medium, and after three days, spheroids were relocated into a 96-well plate containing 1% agarose solution, and 100 µL of fresh medium was added. Spheroid formation and morphology were monitored by light microscopy every day and used for experiments when their diameter was about 350 µm.

### 3.5. Confocal and Thunder Microscopy

For both techniques, Hs578T spheroids were incubated for 18 h at 37 °C both with fluorescent rhodamine B bare- and HSA-modified NPs* at 200 µg/mL, while MCF10a spheroids were incubated for 18 h at 37 °C only with HSA-NPs* at the same concentration and analysed by confocal microscopy and by light fluorescent microscope equipped with Leica Thunder^®^ Imaging System. The uptake of DOX@NPs was tested only with confocal microscopy in Hs578T spheroids treated for 3 h, 6 h, 16 h, and 24 h at 37 °C with DOX@NPs at 200 µg/mL (with a DOX amount of about 5.2 µM). In confocal microscopy analysis, spheroids were carefully transferred into a 24-well plate and fixed with paraformaldehyde (PFA) 2% in PBS (*w/v*) for 1 h at RT. Cell nuclei were stained with Hoechst 33342 at 1.5 µg/mL in PBS 1× for 90 min at RT. Spheroids were then transferred on microscope slides and analysed. Experiments were carried out on an inverted and motorised microscope (Axio Observer Z.1) equipped with a 63×/1.4 Plan Apochromat or a 20×/0.5 EC Plan Neofluar. The attached laser scanning unit (LSM 700 4× pigtailed laser 405–488–555–639; Zeiss, Jena, Germany) enabled confocal imaging. For excitation, 405 nm and 555 nm lasers were used. Fluorescence emission was revealed by Main Dichroic Beam Splitter and Variable Secondary Dichroic Beam Splitter. Double staining fluorescence images were acquired separately using ZEN 2012 software (Zeiss, Germany, Oberkochen) in the red and blue channels at a resolution of 512 × 512 pixels, with the confocal pinhole set to one Airy unit, and then saved in TIFF format.

### 3.6. Light Microscopy Analysis

Hs578T spheroids were incubated for increasing times (24 h, 48 h, 72 h, and 96 h) at 37 °C with HSA-NPs at 200 µg/mL as control, DOX@NPs at 50 µg/mL (DOX at 1.3 µM), 100 µg/mL (DOX at 2.6 µm), and 200 µg/mL (DOX at 5.2 µM) and with corresponding concentrations of free DOX. Spheroids were observed through light microscopy, and images were acquired using a digital camera (Panasonic Lumix DC-FZ82 Bridge) with 10× magnification.

### 3.7. Cell Viability Assay

For CellTiter-GLO 3D assay, Hs578T spheroids were carefully transferred into 96-well opaque plates and incubated at 37 °C with DOX@NPs corresponding to an amount of DOX of 1.3 µM, 2.6 µM, and 5.2 µM (DOX@NPs at 50, 100, and 200 µg/mL, respectively). The assay was performed after 24 h, 48 h, 72 h, and 96 h incubation with nanoparticles following the manufacturer’s instructions. Luminescence was recorded for 0.25 s per well by a Multilabel Reader. For photothermal (PT) experiments, Hs578T spheroids were treated in low attach plates both with MelaSil_Ag-HSA@DOX (1.3 µM, 2.6 µM, and 5.2 µM of DOX) and MelaSil_Ag-HSA (50, 100, and 200 µg/mL) for 18 h. At the end of the incubation, the medium was replaced with fresh medium, and the spheroids were irradiated at 808 nm CW laser for 5 min, with a mean power density of 3 W/cm^2^. The irradiation system consisted of a custom-made adiabatic box containing a motorised linear stage and a heating plate. The laser light was generated by an 808 nm laser diode and brought into the box through an optical fibre (Thorlabs, Newton, NJ, USA) of 1 mm exit diameter. Continuous laser light illumination was controlled by a laser diode controller (LDC220C Thorlabs) and a temperature controller (TED200C). During irradiation, spheroids were maintained at 37 °C. CellTiter GLO 3D assay was performed 3 h, 6 h, and 24 h post irradiation.

### 3.8. High-Frequency Ultrasound and Photoacoustic Imaging of Hs578T Spheroids

Photoacoustic (PA) and ultrasound measurements were performed by the multimodal imaging platform VEVO LAZR-X (VevoLAZR, FUIJIFILM VisualSonics Inc., Toronto, ON, Canada), which produces PA images co-registered with B-mode echo images. For the acquisitions, Hs578T spheroids were incubated in a low attachment plate with DOX@NPs (50, 100, and 200 µg/mL) for 18 h. At the end of the incubation time, the spheroids were washed, fixed with 4% paraformaldehyde, and then embedded in agar phantoms (1% agar) (Figure S4). The spheroids were studied in the first optical windows, 680–970 nm, measuring the PA spectral response. The results are reported in terms of contrast-to-noise ratio (CNR) and signal-to-noise ratio (SNR) at 700 nm, according to the following formulas:

$$SNR=\frac{{Avr\;PAS}_{DOX@NPs}}{{St.Dev}_{DOX@NPs}};$$

$$CNR=\frac{{Avr\;PAS}_{DOX@NPs}-{Avr\;PAS}_{Bkg}}{\sqrt{{St.Dev_{DOX@NPs}}^{2}+{St.Dev_{Bkg}}^{2}}}$$

### 3.9. Statistical Analysis

Results of the assays are expressed as mean ± SD of three independent experiments. Data are reported as average and SD. The statistical significance of differences among groups was evaluated using analysis of variance through the software GraphPad Prism 9.0. Significance was accepted at a confidence level of 95% (*p* < 0.05).

## 4. Conclusions

This study explored the efficiency of the chemo-photothermal and photoacoustic properties of MelaSil_Ag-HSA@DOX nanoparticles, previously assessed in a 2D breast cancer cell culture, in a 3D breast cancer cell model. The uptake of MelaSil_Ag-HSA NPs and MelaSil_Ag-HSA@DOX NPs and their therapeutic efficacy were investigated in dark conditions and after CW laser irradiation. Based on the obtained results, we demonstrated that MelaSil_Ag-HSA@DOX NPs were specifically internalised via HSA-SPARC interaction also in a 3D model, representing a promising platform for integrated chemo- and photothermal therapy, with the advantage of limiting DOX concentration and time exposure. Furthermore, their detectability exploiting the photoacoustic signal of melanin in a three-dimensional cell model represents a starting point to investigate MelaSil_Ag-HSA@DOX NPs as a new theranostic platform. Therefore, using these hybrid melanin-inspired nanoparticles, owing to active targeting, could represent a good strategy to perform early diagnoses and reduce the adverse side effects of DOX in vivo.

## Figures and Tables

**Table 1:** DLS, PDI, and ζ-potential analysis.

	Size (nm)	PDI	ζ-Potential (mV)
MelaSil_Ag	353 ± 17	0.33 ± 0.1	−22 ± 1.3
MelaSil_Ag-HSA	384 ± 30	0.26 ± 0.07	−27.2 ± 1.8
MelaSil_Ag-HSA@DOX	407 ± 29	0.45 ± 0.09	−17 ± 2.16

## Data Availability

Data is contained within the article and Supplementary Materials.

## Supplementary Materials

The following supporting information can be downloaded at: https://www.mdpi.com/article/10.3390/ijms242417374/s1.
