# Supplementary material for: Hybrid Nanoparticle-Assisted Chemo-Photothermal Therapy and Photoacoustic Imaging in a Three-Dimensional Breast Cancer Cell Model
Source: Int J Mol Sci. 2023 Dec 12;24(24):17374. doi: 10.3390/ijms242417374 (PMC10743567; doi:10.3390/ijms242417374)
Supplement: Supplementary file 1 [file ijms-24-17374-s001.zip › ijms-2722960-supplementary.pdf]

Supplementary Materials

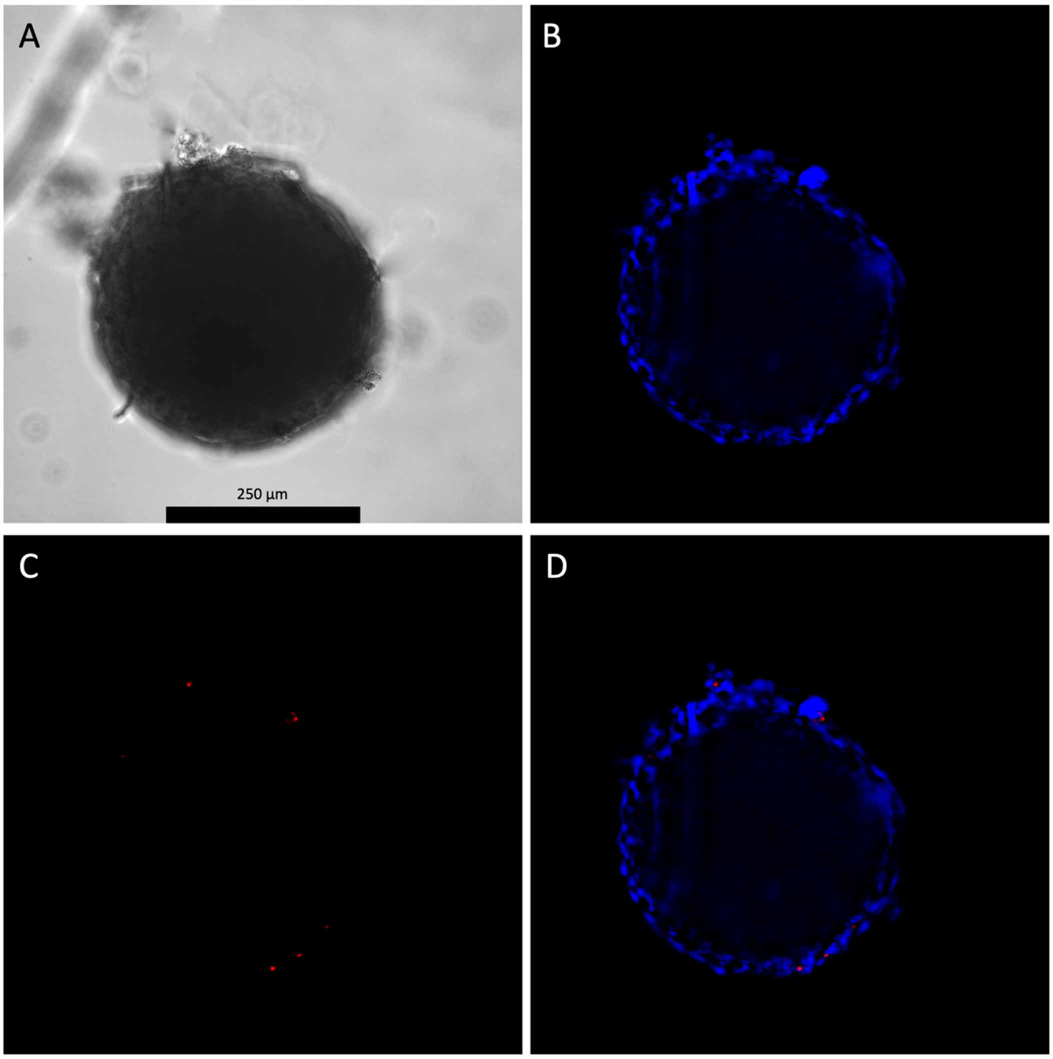

Figure S1 Representative images of Thunder microscopy analysis of untreated HS578T spheroids. A) bright field; B) Hoechst 33342 fluorescence; C) red fluorescence; D) merge. Scalebar 250 µm

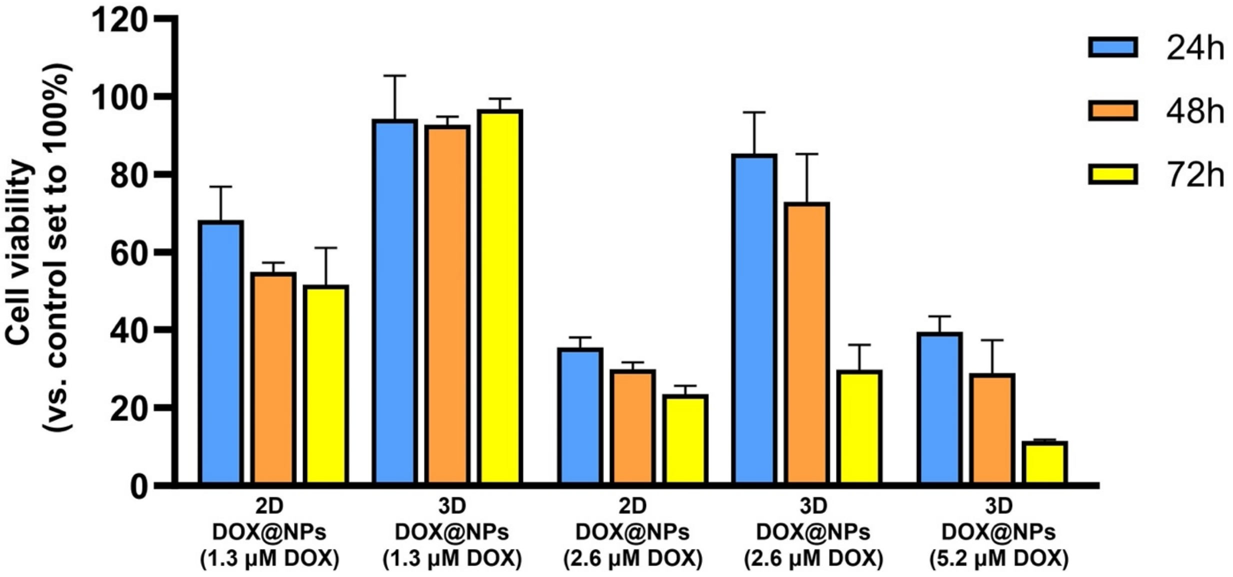

Figure S2 Comparison of DOX@NPs toxicity in 2D and in 3D models

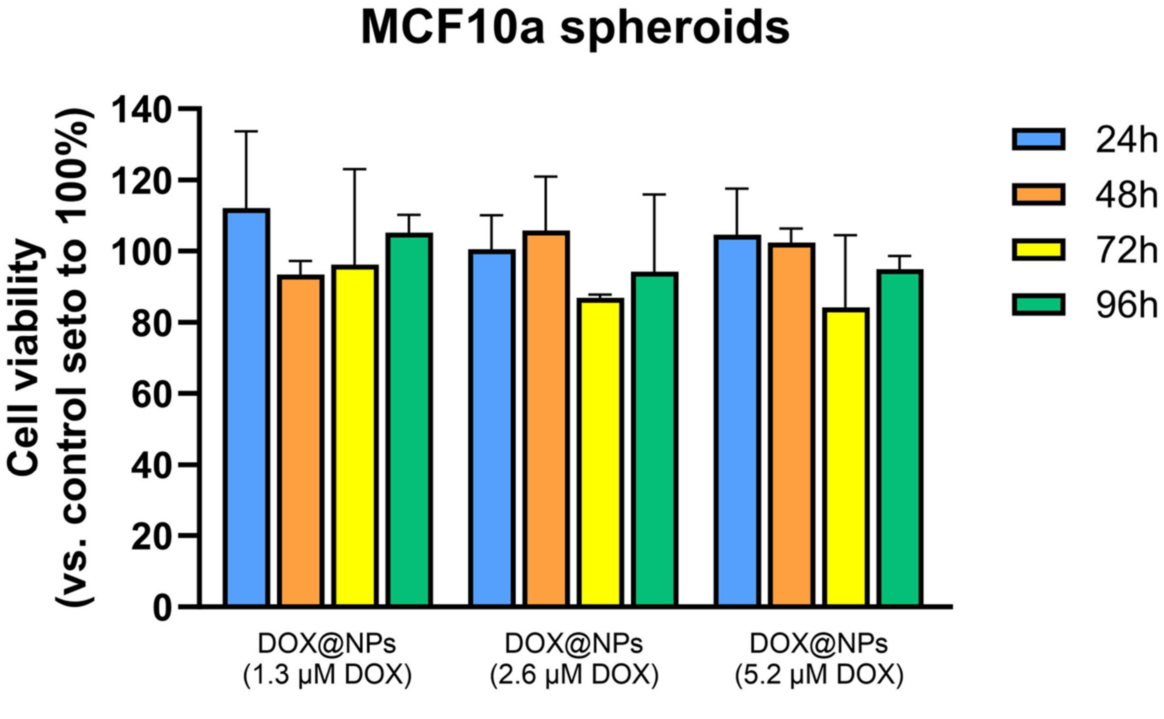

Figure S3 Cell viability assays performed with CellTiter-GLO 3D assay of MCF10a spheroids treated for 24h, 48h, 72h, and 96h with MelaSil_Ag-HSA@DOX NPs with an amount of DOX of about 1.3, 2.6, and 5.2 μM (NPs at 50 μg/mL, 100 μg/mL, and 200 μg/mL, respectively) $p < 0.05$

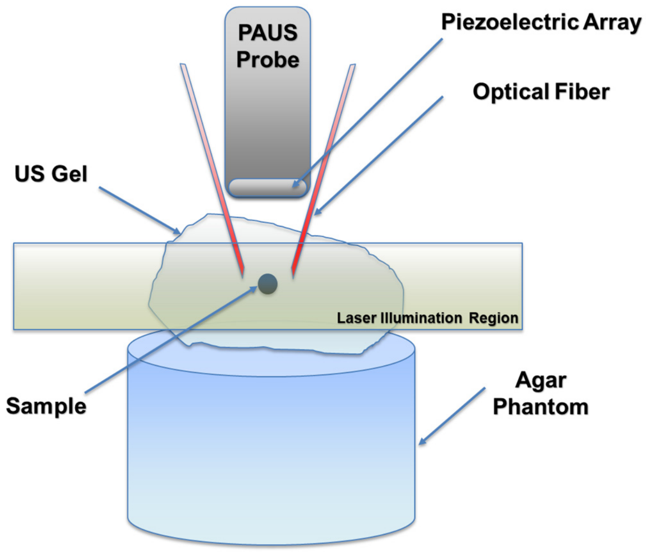

Figure S4 Experimental setup for the photoacoustics multispectral acquisitions of HS578T spheroids
